# Experimental Analysis of the Application of Serverless Computing to IoT Platforms

**DOI:** 10.3390/s21030928

**Published:** 2021-01-30

**Authors:** Priscilla Benedetti, Mauro Femminella, Gianluca Reali, Kris Steenhaut

**Affiliations:** 1 Department of Engineering, University of Perugia, via G.Duranti 93, 06125 Perugia, Italy; pris.benedetti92@gmail.com (P.B.); mauro.femminella@unipg.it (M.F.); 2 Department of Electronics and Informatics (ETRO), Vrije Universiteit Brussel, Pleinlaan 2, 1050 Brussels, Belgium; ksteenha@etrovub.be; 3 Consorzio Nazionale Interuniversitario per le Telecomunicazioni (CNIT), 43124 Parma, Italy

**Keywords:** serverless computing, FaaS, performance evaluation, IoT

## Abstract

Serverless computing, especially implemented through Function-as-a-Service (FaaS) platforms, has recently been gaining popularity as an application deployment model in which functions are automatically instantiated when called and scaled when needed. When a *warm start* deployment mode is used, the FaaS platform gives users the perception of constantly available resources. Conversely, when a *cold start* mode is used, containers running the application’s modules are automatically destroyed when the application has been executed. The latter can lead to considerable resource and cost savings. In this paper, we explore the suitability of both modes for deploying Internet of Things (IoT) applications considering a low resources testbed comparable to an edge node. We discuss the implementation and the experimental analysis of an IoT serverless platform that includes typical IoT service elements. A performance study in terms of resource consumption and latency is presented for the warm and cold start deployment mode, and implemented using OpenFaaS, a well-known open-source FaaS framework which allows to test a cold start deployment with precise inactivity time setup thanks to its flexibility. This experimental analysis allows to evaluate the aptness of the two deployment modes under different operating conditions: Exploiting OpenFaaS minimum inactivity time setup, we find that the cold start mode can be convenient in order to save edge nodes limited resources, but only if the data transmission period is significantly higher than the time needed to trigger containers shutdown.

## 1. Introduction

Internet of Things (IoT) is a popular expression widely used to encompass the many related networking and application aspects. It involves the extension of Internet connectivity to objects beyond traditional computing devices [1]. The continuing advancement of IoT service platforms has been progressively driven by the inclusion of new and relatively complex components. They allow the implementation of different categories of services, characterized by extremely variable and disparate network and computing resource requirements. Sometimes, these categories coexist on the same distributed platform. For example, measurements collected by sensors in the city for environmental monitoring can be used for traffic control, surveillance, pollution control or even for tourism management. Hence, the collected data will be integrated into various applications, characterized by different quality of service requirements, number of information flows, volume of data exchanged, data protection requirements etc. To face these issues, a popular strategy consists in leveraging the so-called Edge Computing [2,3]. An Edge Computing architecture is a distributed architecture of micro data centers, capable of locally storing and processing data, as well as being able to transmit these raw or processed data to a centralized collector or a cloud storage repository. The concept of Edge Computing is related to the so-called Fog Computing. While the boundary between the two technologies is blurred, edge computing is typically regarded as a service implemented either on the devices to which the sensors are attached or very close to it, as at a gateway node of the local sensor network. Fog computing means a service that takes place in a local network, close to sensors. Taking advantage of its fast deployment and flexibility, fog computing can serve IoT networks even when located in critical geographical areas, as shown in [4], where authors consider scenarios such as underwater monitoring and military deployment. However, the definition of edge computing is quite loose. For instance, in ETSI it is associated with the concept of Multi-access Edge Computing (MEC). In [5], the authors discuss some potential deployment options for edge nodes implementing MEC in 4G/5G networks, encompassing all the scenarios in which the MEC platform installation point ranges in locations between the base station itself and the mobile core network, immediately before the Internet gateway. Thus, in general running applications in edge computing means deploying them close enough to the end users in order to save transmission resources with respect to a mere cloud deployment, by keeping data locally. Thus, the main features of Edge Computing include location awareness, dense geographical distribution, and limited amount of computing and storage resources available [6,7]. The analysis shown in this article concerns the scarce availability of resources and its management by using advanced virtualization techniques, for services whose latency requirements are not very strict.

Edge computing is therefore an alternative to the sole use of centralized data centers, which have long since revealed their drawbacks for significant data volumes management and data protection. Clearly, moving the workloads to the cloud allows to significantly reduce the production costs and to take advantage of the dynamic access to virtually unlimited storage and computing resources. However, not all applications are portable to the cloud. In particular situations, the stochastic nature of the access to services in the cloud is unsuitable for steadily supporting continuous flows of data from sensors and for timely offering an IoT service. Another issue is related to data protection policies enforced by the current legislation. To overcome these problems, Edge Computing is appearing in many different application areas. Nevertheless, the critical factor of Edge Computing is the limited amount of available resources. For this reason, it is strategic to resort to resource management technologies that obtain optimal resources usage to support as many users as possible.

In this paper we explore the suitability of the so-called serverless computing [8] for deploying IoT-based services. It leverages the success of virtualization technologies, based either on virtual machines (VMs) or containers, and has stimulated cloud industry to pursue a new architectural design exploiting virtualization to deploy platform as-a-service. Any service platform must satisfy some general requirements to effectively support virtualized node deployment [9]. It must be easily scalable with minimal configuration and management effort, making efficient use of resources while maintaining a secure environment in which services are isolated from each other. The idea behind serverless computing is that computing resources, along with their configuration and management, are dynamically provisioned at run-time, through an event-driven allocation control, typically referred to as function as a service (FaaS). FaaS is intended to be different from software as a service (SaaS) because of its event-based architecture and auto-scalability, emphasizing its virtual independence from the underlying servers (i.e., serverless). This is in contrast to traditional methods in which the needed resources are planned during application design and provided during the deployment phase. Indeed, the name serverless computing highlights that application design does not include production servers but can only focus on the application code, organized through individual microservices executed in microVMs or containers. This is the key difference between FaaS and the other cloud service models, such as Infrastructure as a Service (IaaS), Platform as a Service (PaaS), and Software as a Service (SaaS). Instead of deploying a whole platform or an application in the cloud servers, by using FaaS just functions are required, as components of complex applications. Such functions can be loaded as virtual containers when needed, on demand, and possibly in parallel, without any need for controlling application deployment processes at the operating-system level. This means that containers are dynamically scheduled in the hardware infrastructure maintained by cloud providers. This approach allows service designers and users to only focus on the application logic, without having to deal with server management and autoscaling functions, which are intrinsically included in the FaaS service.

Through the use of advanced virtualization technologies, FaaS can ensure that the volume of resources consumed by an application is dynamically controlled and is tailored to the actual computing needs. This is clearly a valuable feature for short and on-demand tasks. In fact, in IoT systems, serverless computing allows to integrate data produced by a variety of devices, that are silent for most of the time, and transmit short data packets periodically or randomly. These events can trigger the instantiation of the necessary functions. Those functions will be included in the service logic only when needed, allowing efficient resource usage and associated cost savings. However, this approach may cause some latency issues. These are analyzed in this paper, by a number of experiments that have as specific purpose to identify when the serverless model is convenient, emphasizing the distinction between the cold start and warm start for application development. The presented experiments cover various metrics for the analysis of serverless platforms, considering time latencies, resources usage, messages losses and traffic volumes. We evaluate the convenience of a cold start deployment to save resources in a node with low capabilities.

In summary, the objective of this paper is twofold. We first discuss several serverless computing technologies, that can be used for addressing the challenges presented above. Then we present the implementation of a serverless platform which includes typical IoT service elements. Performance is evaluated in terms of resource consumption and latency. This evaluation considers both deployment modes, warm and cold start, implemented through OpenFaaS [10], a well-known open-source FaaS framework. It allows to evaluate the aptness of each of the modes under different operating conditions. The paper is organized as follows. In Section 2, we analyze related work. Section 3 presents the main features of serverless computing and some recently introduced technologies. We illustrate a baseline IoT service architecture based on these technologies and use it for executing experiments. The results are analyzed in Section 4. Finally, Section 5 reports our conclusions and perspectives for future work.

## 2. Related Work

This section presents some recent contributions to serverless computing. A discussion of serverless techniques is given in [11]. The authors qualitatively compare several open-source technologies for serverless computing and quantitatively evaluate results of their application that makes use of those technologies. The analysis is interesting, but is not aimed at determining the conditions of when to apply serverless computing in edge networks.

An extensive experimental analysis of serverless platforms is presented in [12]. The authors focus on the performance of popular open-source serverless platforms for several key applications and design choices. They focus on throughput and latency issues, in particular when autoscaling is needed. Their analysis sheds some light on the capabilities of serverless computing, although its suitability for edge-based applications, which is the focus of this paper, is not covered.

In [8] the authors present a general description of serverless computing and its relevant programming model, pointing to Function-as-a-Service (FaaS) platforms as the concretisation of serverless computing principles. As a future promising research activity they identify the application of the serverless model at the edge as natural connection between serverless functions and Edge Computing, in particular for IoT.

Paper [13] shows preliminary results related to the use of WebAssembly as an alternative method for running serverless applications. Several case studies were implemented in a local Edge Computing-based infrastructure, which relates the paper to our analysis. While the experimental results show that WebAssembly can be a viable alternative to the use of containers in serverless platforms, no comparison with other container-based serverless technologies such as OpenFaas is shown and further analysis is necessary.

Experimental assessment of serverless computing technologies deployed on a Kubernetes cluster is reported in [14]. The authors analyze the status of several open source serverless computing technologies, namely Fission, Kubeless, OpenFaaS and OpenWhisk. The latter, Apache OpenWhisk, is not considered in the performance evaluation due to its low dependence on Kubernetes for orchestration tasks. Their study was useful to identify some key aspects of OpenFaaS, a technology also used in our experiments, highlighting its easy extensibility and good performance across different scenarios. In particular, it shows the greatest flexibility for supporting multiple container orchestrators and highest adoption rate among the analyzed serverless frameworks. The analysis does not include service deployment in edge networks, which is mentioned as future research.

Serverless computing is also discussed in [15], where the authors focus on programming model and DevOps challenges. They wonder when it is convenient to use this implementation and deployment model, but leave this question for future analyses.

In [16], authors analyse factors that influence the perceived duration of the cold start by performing a benchmark on AWS Lambda and Microsoft Azure Functions with 49,500 cloud function executions. They assess the cold start from a user point of view, as the chosen platforms report only a fraction of cold start overhead, showing an higher than expected gap between functions developed in compiled languages and the ones developed in interpreted languages. Further investigation of cold start impact on other well-known FaaS platforms like OpenFaaS, which is one of the contributions of this work, still has to be conducted. General pros and cons of the serverless computing model are presented in [17]. The authors describe the main issues affecting the performance of serverless computing platforms and present some experimental results. Their investigation makes use of serverless functions made available in commercial cloud for three specific case studies, being machine learning based model training, live predictions, and classification of batching inputs. While the presented experiments are interesting, the usage of a single commercial platform hinders the general nature of the results. In addition, deployment in edge networks with limited resources was not considered.

A serverless edge platform based on OpenWhisk is presented in [18], along with different application scenarios and an evaluation in terms of memory footprint, latency, throughput, and scalability. Results show the benefits of serverless architectures, but a performance evaluation of the platform in cold start versus a warm start approach is not provided.

In [19], a serverless approach used to manage IoT traffic is analyzed. The authors developed serverless function chains, based on Knative [20], a Kubernetes-based platform to deploy serverless workloads, to manage home monitoring and agricultural applications. The authors used the CloudLab [21] infrastructure to simulate IoT traffic for an Edge Computing scenario. However, no details on the used hardware resources are given. The paper shows potential resource usage benefits due to serverless computing. However, the Knative design introduces significant overhead due to management components, such as the queue-proxy sidecar container in the cold start usage. No comparison with the warm start deployment on the same architecture was shown. In this work, we analyse the benefits of a serverless IoT application deployment focusing on the differences among two different approaches, cold and warm start mode. For this work, we consider the highly adopted open-source serverless framework, OpenFaaS, of which cold start and warm start deployment performances have not been analysed before.

## 3. System Architecture

**Figure 1:**
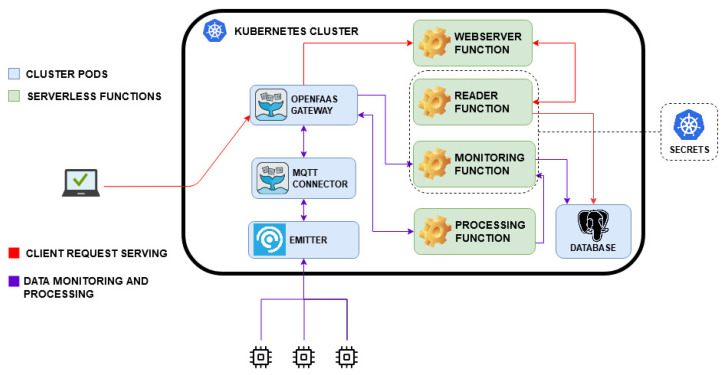
In the test platform, serverless functions are deployed in a Kubernetes cluster using the OpenFaas framework [10] to monitor, process and expose via webserver external event messages, published on an Message Queuing Telemetry Transport (MQTT) Emitter broker (FaaS stands for Function-as-a-Service) [25].

In this work, we analyze the achievable performance of an event monitoring and processing serverless platform. This system, shown in Figure 1, is suitable for IoT scenarios where the rate of exchanged messages is relatively low. This way, the computing effort needed for occasional monitoring, storing and processing of data can make the serverless approach convenient. For example, the platform presented in what follows could be used for implementing video-surveillance applications, which typically generate sporadic transmissions. IoT empowered video-surveillance deployment is having an unprecedented growth: according to a report by the Information Handling Services there is a camera installed for every 29 people on the planet and this number will increase by 20% in the coming years [22] Another example consists of applications for telemedicine and smart health, where patient monitoring requires data exchange only periodically, with a period of several minutes or even hours, and to report emergencies. This is the case of Remote Patient Monitoring (RTM), which ensures that patients with chronic diseases, such as heart and diabetic conditions, get periodically monitored at home, alerting the doctor when it detects a problem with the patient. In fact, Internet of Medical Things (IoMT) is a growing market, expected to reach $332 Billion by 2027, according to forecasts by Allied Market Research [23]. The serverless functions have been developed by using OpenFaaS [10], in order to manage and coherently evaluate a cold start approach, taking advantage of the OpenFaaS lower keep-alive time (1 min), compared with other open-source FaaS implementations (e.g., OpenWhisk uses a 10-min period [24]).

OpenFaaS is a Cloud Native Computing Foundation (CNCF) open source serverless framework. It allows developers to define and use templates of different languages to create and build serverless functions. It relies on Docker images and Kubernetes control plane to run applications, providing fail-over, high availability (HA), scale-out and secret management. In the OpenFaaS framework, the OpenFaaS gateway, which is similar to a reverse proxy, is in charge of exposing and managing the function pods, offering a REST API for all interactions. Taking advantage of OpenFaaS trigger functionality, this serverless system is subscribed to a Message Queuing Telemetry Transport (MQTT) Emitter broker in order to monitor messages from the outside. For this test, we developed four serverless Python functions, using Flask HTTP framework [26]:

- Monitoring Function: This function monitors the event signalling messages coming from the MQTT Emitter broker, stores them in a PostgreSQL database and sends them to the processing function when messages metrics exceed a defined alarm threshold. Once received from the processing function, it stores the processed data in a dedicated table in the database.
- Processing Function: This function receives the alarm event messages, classifies them using a pre-trained machine learning algorithm and sends data back to the monitoring function.
- Webserver Function: This function can be invoked by a platform registered user to monitor the stored messages, both labeled and unlabeled.
- Reader Function: This function is responsible for fetching the stored messages from the database.

**Figure 2:**
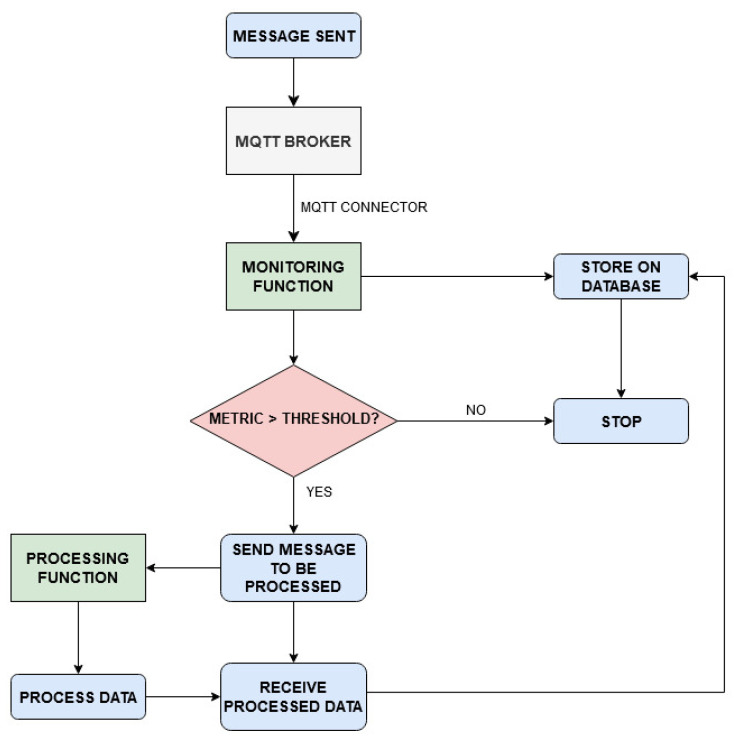
Flowchart for data monitoring and processing procedures.

**Figure 3:**
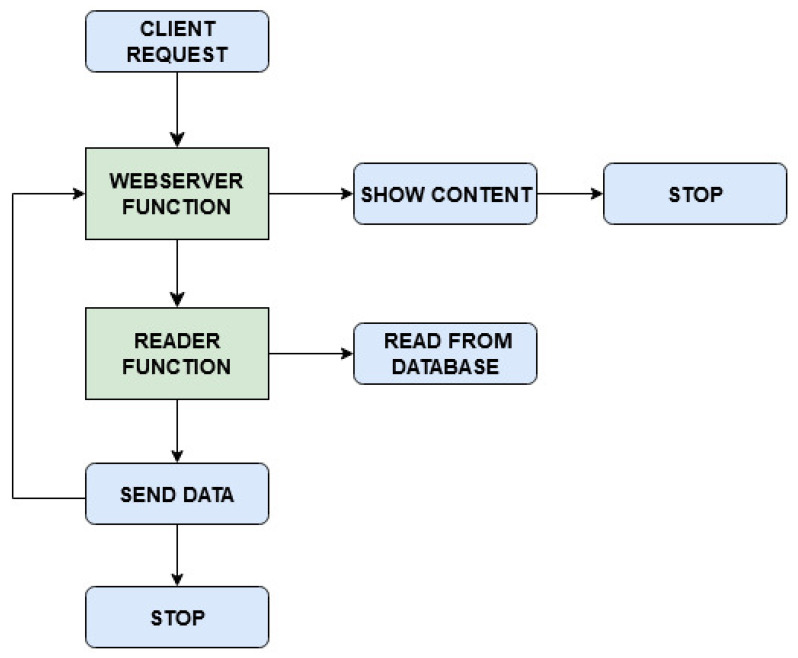
Flowchart for client request serving procedure.

**Figure 4:**
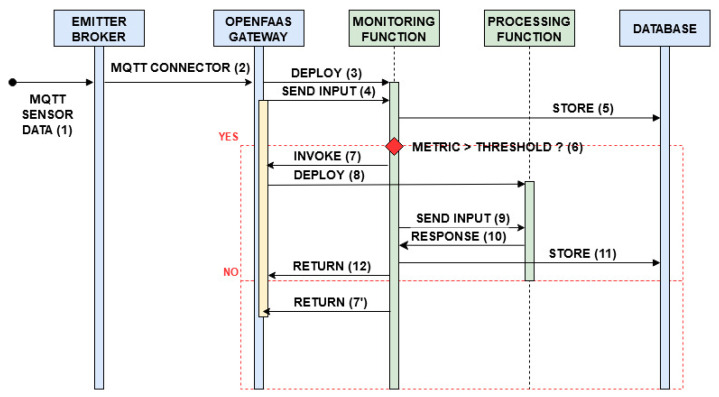
Sequence diagram for data monitoring and processing.

**Figure 5:**
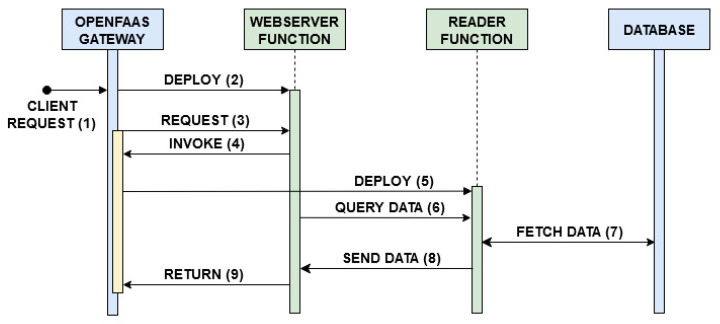
Sequence diagram for client requests serving.

All requests arriving from outside the cluster, destined to the Emitter broker and to the OpenFaaS gateway are filtered by a firewall. The read-write access to the PosgreSQL database is managed and secured by the use of Kubernetes secrets. Two main processes are tested: data monitoring with occasional event processing, described in the flowchart shown in Figure 2, and client request serving, Figure 3. The corresponding sequence diagrams for both the procedures are shown in Figure 4 and Figure 5:

- Data Monitoring and Processing (Figure 4): When a message is published in a topic (1), the Emitter broker, via OpenFaaS native MQTT connector (2), invokes the serverless monitoring function subscribed to that topic, sending the message of interest as input. The OpenFaaS Gateway deploys the needed pod for the monitoring function (3) and, as a proxy, redirects to it the input from the broker (4). This function stores the received data in the database (5) and analyzes a specific metric entry in the message (6). If this metric is lower than the defined alarm threshold, the function ends (7’). If not, by invoking the processing function via the OpenFaaS Gateway API (7), the monitoring function triggers its deployment (8) and finally the message is sent to the serverless processing function (9). After having labelled the input using a machine learning algorithm, this function sends back the processed data to the monitoring function (10) which saves the labelled message in the database (11) and ends its process (12).
- Client Requests Serving (Figure 5): When a client requests a view of processed and/or simply monitored data (1), the serverless webserver is deployed (2) by the OpenFaas gateway. The request is redirected from the gateway to the webserver (3). This function, in order to requests database entries from the serverless reader function, invokes its deployment (4,5). Once deployed, the serverless reader function receives the query (6), fetches the data from the database (7), returns the entries to the webserver (8) and stops. When the content is shown, the webserver function ends (9).

The system under study was built and evaluated following two different approaches, in compliance with the serverless paradigm: i.e., deploying the four aforementioned functions with warm start or with cold start. As shown in Figure 4 and Figure 5, after receiving MQTT sensor data or a client request, the OpenFaaS Gateway initially deploys the monitoring function (3, Figure 4) or the webserver function (2, Figure 5). If necessary, subsequently the monitoring function invokes the processing function through a request to the OpenFaaS Gateway (8, Figure 4), analogously for the webserver function in order to get data from the reader function (5, Figure 5). In the warm start case, after the first deployment by the OpenFaaS Gateway, each serverless function pod remains active, waiting for other calls. Meanwhile, in the cold start case, each serverless function pod is deployed and terminated after a period of inactivity $T_{idle}$, in order to save resources at the expense of increased latency. These latencies are caused by repeating each function pod deployment (as in 3,8 in Figure 4 and 2,5 in Figure 5).

The cold and warm start operating modes, considered in the following analysis are exhaustive for the serverless context. In fact, the warm approach is comparable to a classic deployment whereas the cold one implements the extreme serverless model. We emphasize that it would be useless to present a case study with the services implemented in the host operating system or in a virtual machine since this work aims to be a serverless platform analysis. Moreover, time performance of an application implemented in a host operating system or in a virtual machine would be similar to that of the warm deployment mode, aside from a single initial transient.

## 4. Performance Evaluation

### 4.1. Test Environment and Research Methodology

In this section, we analyze the performance of the proposed architecture, based on the serverless paradigm. Implementation is based on the OpenFaas platform [10], the open-source serverless framework with the highest adoption rate as shown in [14], over which two main implementation architectural alternatives are implemented: cold start and warm start.

The test-bed edge infrastructure consists of a host equipped with sufficient computing resources (4 CPU cores, 4 GB of RAM, 250 GB of storage) to serve the offered processing peak load, as we have verified experimentally. The amount of resources used for the testbed are in line with the indications provided in many papers [6,7,27], which consider as edge node even single board devices, such as a Raspberry PI, or a notebook/desktop with medium processing capabilities. In this way, the computing capacity of the host node does not have any significant impact on the collected metric values. In order to discriminate which implementation alternative is more suitable in the considered operational conditions, we run different experiments, setting the timer for cold start to the minimum allowed value in the standard OpenFaaS configuration, i.e., $T_{idle}$ = 1 min. This way we can evaluate both service time (Section 4.2) and resource usage (Section 4.3) when the platform operates close to the switching point between always on (warm start) and continuous on-off (cold start).

To measure the service time, we introduced a timestamp in the payload of the MQTT messages published by each sensor. We also recorded a timestamp when the message was stored in the database. By computing the difference between these timestamps, we determined the service time. Sensors are emulated by a set of processes running in the same edge node hosting the serverless platform. This way, we avoided any synchronization issue between the two timestamps, as well as any further delay due to the network, since they are issued by the same host. As such, it is possible to analyse the serverless suitability without any other disturbing effects.

We also measured the time of the web query response, which provides the requesting client with the latest 30 measures. In this case, the service time was estimated directly by using a periodic curl process running in the same host. In order to extract more general results from the current set of experiments, we plot the service time as a function of normalized transmission period, that is the ratio between the actual data transmission period from sensors ($T_{sensor}$) and the idle time configured in OpenFaas $T_{idle}$. It represents the period of inactivity after which the serverless function, running in the pod, is terminated.

In order to measure the resource consumption associated with the two deployment options, we used Prometheus [28] to collect resource usage statistics. We selected Prometheus since it is a well-known open-source monitoring system, widely used in Kubernetes production environment and integrated in the OpenFaaS stack [29]. In fact, Prometheus allows getting usage data for per Kubernetes pod running a different function.

### 4.2. Service Time

The offered service consists of two main functions, namely the monitoring function, which is executed only when a sensor measurement is sent, and the processing function, which is selectively triggered by the monitoring function according to the result of a comparison between the received data and a predefined threshold. We report the service time for both functions.

**Figure 6:**
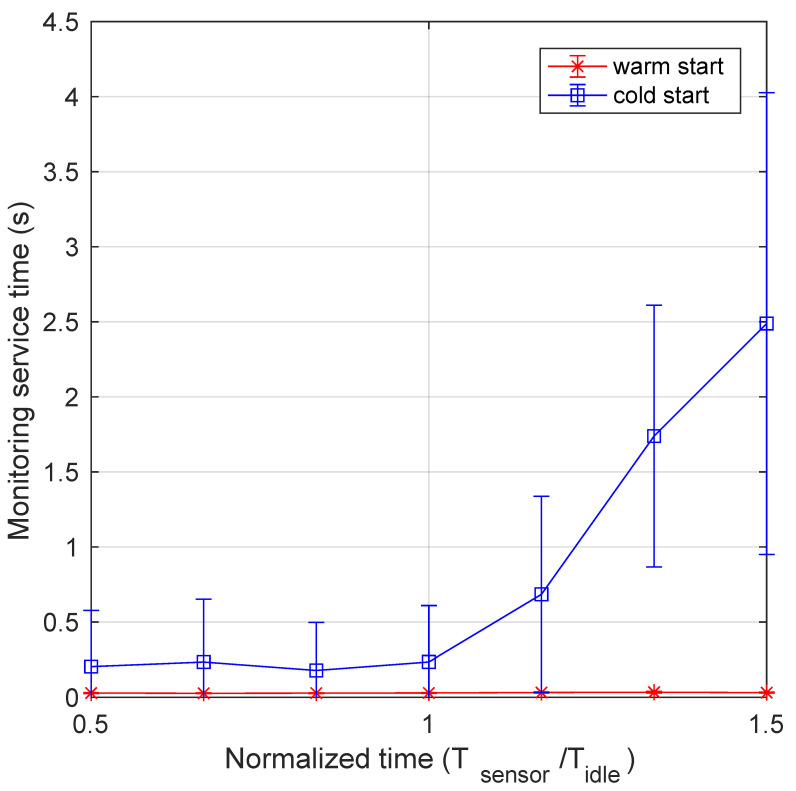
Monitoring time as a function of the normalized transmission period for both cold and warm start.

**Figure 7:**
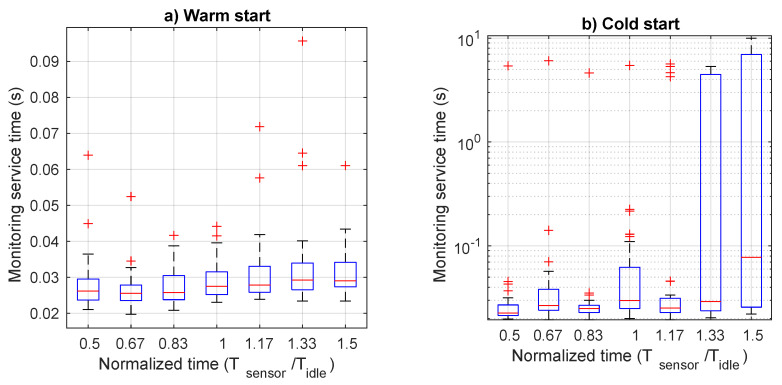
Boxplot of monitoring service time as a function of the normalized transmission period: (**a**) warm start and (**b**) cold start.

Figure 6 shows monitoring time as function of normalized data arrival period ($T_{sensor}/T_{idle}$, for both cold start and warm start modes, with the relevant 95% confidence intervals. From the graph it is clear that running the monitoring function in the cold start mode implies a significant penalty in term of response time. In fact, whereas it is well below 0.5 s for the warm start mode, independently on the arrival rate, quite different values are observed for the cold start. Until $T_{sensor}$ is lower than $T_{idle}$, the service time is just slightly higher than the value in the warm start mode. This is because it suffers from sporadic termination of the function and subsequent restart due to arrival of data to be managed. However, when the data arrival rate increases, shutdown and restart of the monitoring function is always more frequent, and has a significant impact on service time. In particular, it increases with a constant rate up to an average value of 2.5 s. To have a deeper insight of what happens for each value, we can inspect more detailed data in Figure 7, which presents the boxplot of monitoring time for both warm and cold start. In this figure, we observe a nearly stable time for the warm start case as opposed to the high variability of the cold start case. In fact, outliers for the warm start are limited to 1 s, whereas the upper bound for the cold start is 10 s. When the normalized data generation period reaches 1, also the 75-percentile starts to increase, although the median value remains very low, until the abscissa reaches the value of 1.5. In this case, the 75-percentile increases up to 7 s, whereas the median value up to 0.8 s. In order to better display the values for the cold start case, we use a logarithmic scale on the ordinate axis.

**Figure 8:**
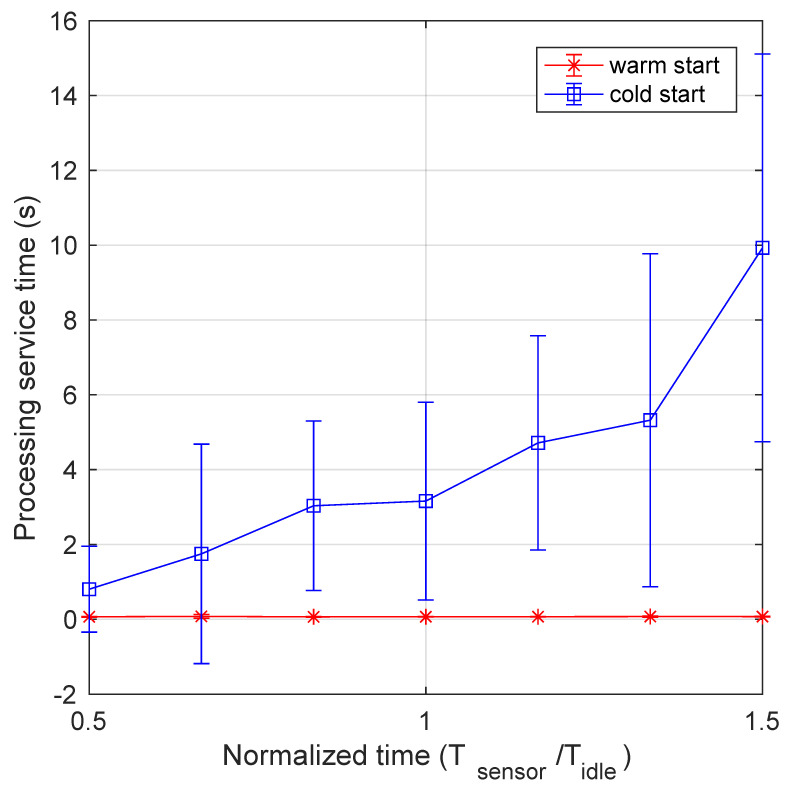
Processing time as a function of the normalized transmission period for both cold and warm start. The impact of pods termination and subsequent restart in cold start mode is manifest.

**Figure 9:**
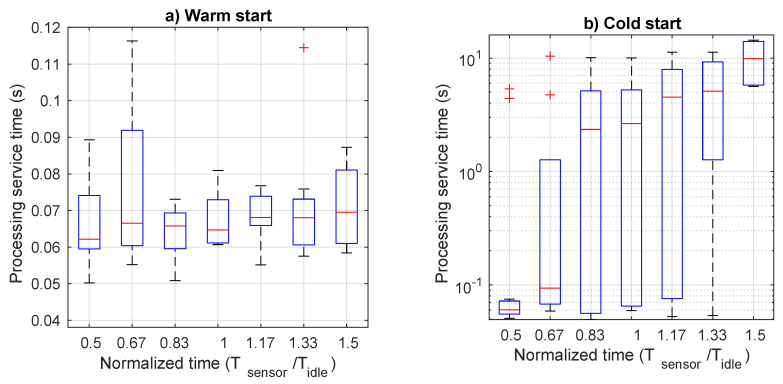
Boxplot of processing service time as a function of the normalized transmission period: (**a**) warm start and (**b**) cold start.

Figure 8 depicts the mean processing time vs. the normalized data generation period $T_{sensor}/T_{idle}$. It is interesting to see that, although the warm start curve remains nearly flat, the cold start curve steadily increases starting from the minimum value of the abscissa. This behavior can be explained by recalling that the processing function is not invoked for each data packet arriving at the system. Instead, the monitoring function invokes it upon comparing data values with a predefined threshold. Thus, when the mean packet arrival rate, to be elaborated by the processing function, increases, the probability to find it still running also increases, and the processing time is inferior. Conversely, when the mean time between two function invocations becomes larger, it is more likely to find the processing function not ready to process data, and it has to be restarted, with consequent higher service time. It is worth noting that, as described in Section 4.1, the processing time includes the time from packet generation to its insertion in the database, thus it also includes the service time of the monitoring function. This can be appreciated in the more detailed view of data provided by the boxplots presented in Figure 9, which can be compared with Figure 7. Again, the values of the processing time in the warm start case are stable and this is evident also in their distribution for different abscissa values. Since their distribution is somewhat more variable, outliers are nearly absent. Instead, the high variability present in the cold start mode is evident, also for abscissa values significantly lower than 1. It is interesting to note that, for an abscissa value of 1.5, both mean and median values of the processing time is around 10 s.

**Figure 10:**
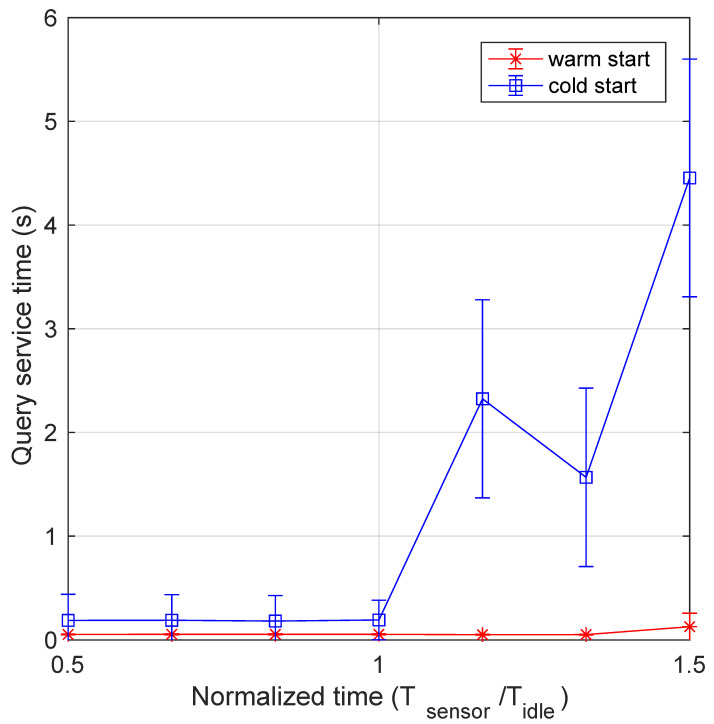
Query time as a function of the normalized transmission period for both cold and warm start.

**Figure 11:**
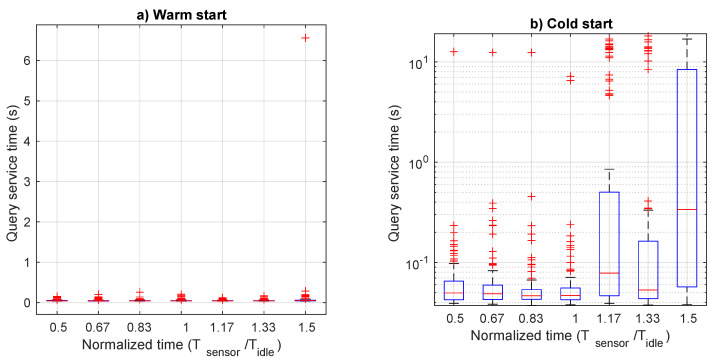
Boxplot of query service time as a function of the normalized transmission period: (**a**) warm start and (**b**) cold start.

Finally, we consider the service time of the web query function, which is expected to run periodically, with values that can be quite relaxed. These queries are used to update data in remote control position, which may need to monitor the last 30 samples. As specified in Section 3, this service time is the result of the execution of two functions: the web server, which is invoked when a remote REST request arrives, and the the subsequent DB reader. Once this second function provided the requested data to the first one, this can reply the remote web client. Since we were interested to the total response time, we measured this time on the client issuing the web request. As for the previous service times, Figure 10 shows aggregate average values, whereas the Figure 11 includes more details through boxplots. Again, performance of the warm start case is very good for all load values, with average and median values lower than 0.05 s and outliers always below 0.2 s. Instead, in the cold start case, we observe a behaviour similar to that of the monitoring service time. In particular, a stable service time is observed up to values of $T_{sensor}\leq T_{idle}$, then increasing up to average values of 4.5 s, with a 75-percentile value larger than 10 s.

It is useful to comment on the suitability of these service times with respect to service requirements. It is clear that for classes of service where the service time has to be upper bounded by a challenging threshold value, such as in the order of some (tens of) ms, the cold start option is not suitable. In fact, if the function that has to manage the arriving data is not active, it is necessary to wait for its restart before the processing can occur. To prevent this problem, cloud service providers offering services implemented with the serverless approach, such as Amazon, Microsoft of Google usually have a pool of identical functions always active. This will minimize the service disruption associated with a function to be started (see, e.g., [17]. The main benefit of serverless adoption is in the resource saving due to the built-in autoscaling property of the FaaS paradigm, which seems the perfect fit for the pay-as-you-go business model. Note that in some cases where such bounds can absolutely not be violated, also the warm start approach could be problematic, at least for the first invocation.

Said this, not all services are equal. If the service time requirement is quite loose, e.g., a few seconds as the observed phenomena has a larger time scale, not only the warm start option is a very good fit, but also the cold start one can be used. In the next subsection, we investigate if the adoption of the cold start implementation can have any real benefits in terms of resource consumption, which is (one of) the metric(s) of highest interest in the serverless computing world.

**Figure 12:**
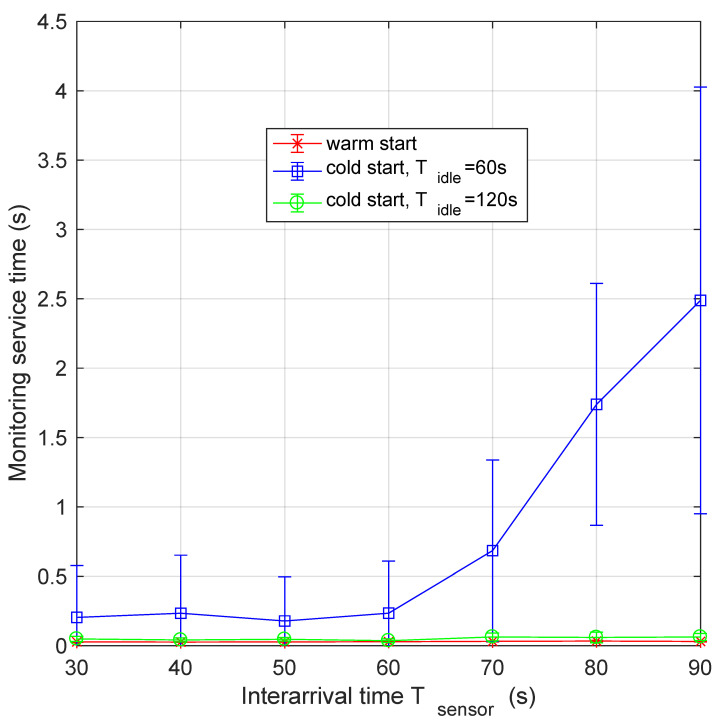
Monitoring time as a function of data interarrival period for both warm start and cold start, with $T_{idle}$ equal to 1 and 2 min. As shown by the green curve, for $T_{idle}$ values larger than data interarrival time, pods are always active, precluding cold start mode applicability.

In order to evaluate the effect of using different $T_{idle}$ values on the system performance, we executed some experiments by using $T_{idle}$ values larger than 1 min. Figure 12 shows the monitoring service time for both warm start (red curve) and cold start, with $T_{idle}$ set equal to 1 and 2 min (the blue and green curves, respectively). We actually used even larger $T_{idle}$, but since the relevant performance is identical to that obtained using a value of $T_{idle}$ = 2 min, we do not include the relevant curves for figure readability. On the abscissa, we report the values of $T_{sensor}$ expressed in seconds. The main comment is that, until $T_{sensor}\leq T_{idle}$, the performance of the system is very close the warm start service deployment. For $T_{idle}$ = 1 min, the switching point happens at exactly $T_{sensor}$ = 60 s. For larger values, the monitoring latency increases up to maximum values in the order of few seconds (see Figure 7). If the data interarrival time is always shorter than the timer triggering the function shutdown, this function remains always active. Thus, its behaviour can be assimilated to the case of warm start, as expected. Thus, the performance achievable by the other platforms, such as OpenWhisk, which allow setting a larger minimum value of $T_{idle}$ (e.g., in OpenWhisk $\min\{T_{idle}\}=10$ m [24]), is similar to that of the green curve in Figure 12.

**Figure 13:**
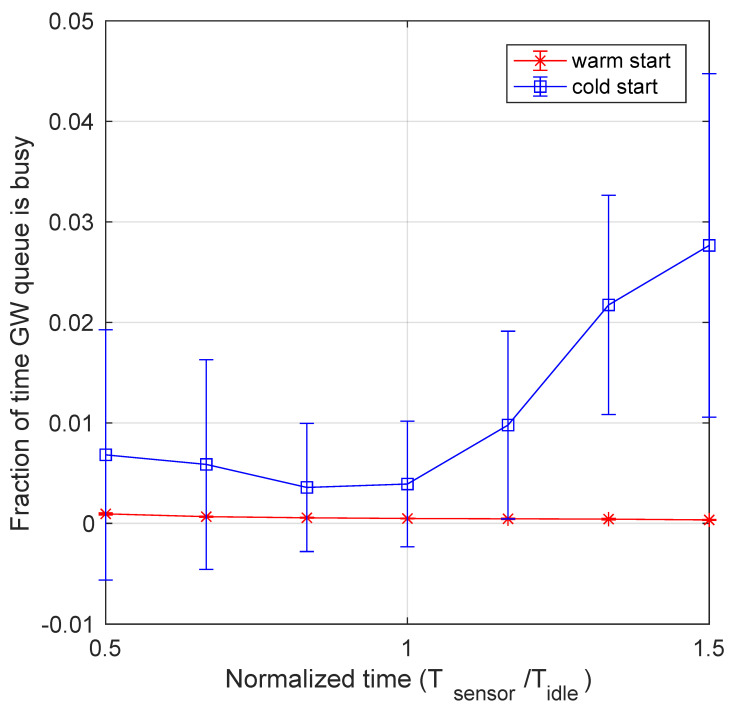
Fraction of time the OpenFaaS Gateway queue is busy as a function of data arrival rate for both cold and warm start. A cold start mode deployment leads to a growth of queued messages waiting for the function to become active and handle data.

Figure 13 shows the fraction of time the queue of the OpenFaaS Gateway handling incoming messages is not empty, i.e., busy, measured over the total experiment duration, as a function of the normalized data generation period $T_{sensor}/T_{idle}$. The figure shows the system behaviour for both the cold and the warm start options. The first comment is that, with the considered data arrival rates, the gateway queue managing incoming messages is inactive most of the time, and only for a very small fraction of time it becomes busy. In these cases, the number of messages present in the gateway queue never exceeds 1, and we never registered packet losses. In the warm start option, the comment is straightforward: as the data arrival rate increases (i.e., $T_{sensor}$ decreases), the fraction of time the gateway queue is busy to manage the incoming messages increases as well. A less obvious behaviour can be observed in cold start option, where two distinct contributions can be observed. The first one is that, for very small arrival rate (i.e., large values of $T_{sensor}$), most of the time the pod running the desired function is not active and the message remains in the gateway queue until that function is able to handle the message. A slight increase of the arrival rate improves this behaviour, since sometimes the pod running the desired function results still active when the next message arrives. Thus, the gateway is faster in delivering the message to the intended function, and the fraction of time when its queue is busy decreases. By further increasing the data arrival rate (i.e., $T_{sensor}$) another behaviour emerges, analogous to that of the warm start option: The higher the volume of messages, the higher the time the gateway queue is busy to handle them, thus the fraction of time during which the gateway queue is busy slightly increases.

### 4.3. Resource Consumption

In this subsection, we illustrate the resource consumption associated with the two deployment options, namely the cold start and the warm start. The different performance figures we propose are the amount of CPU, RAM, and the traffic volume to be exchanged for implementing the example service. These metrics represent an indirect evaluation of the overall complexity of proposed scheme, which has an impact on both computing and network resource consumption.

**Figure 14:**
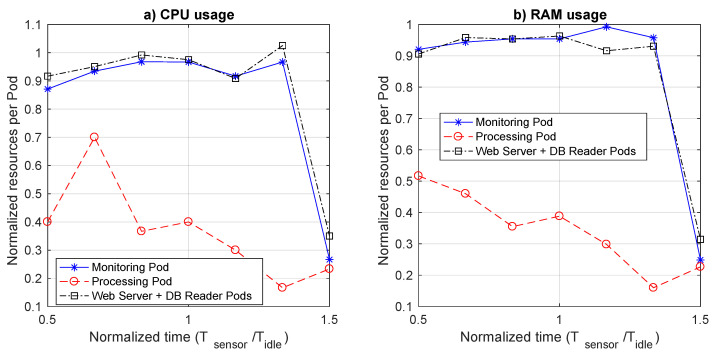
Normalized resource consumption in terms of CPU (**a**) and RAM (**b**) of the cold start option with respect to that of the warm start one, as a function of the normalized data arrival period.

Figure 14 illustrates the resource consumption in terms of CPU and memory (RAM) usage for both the warm and the cold start options. To directly compare these two options, we show a normalized resource consumption. In particular, for the CPU we measured the CPU time during each experiment, for both the warm and the cold start cases, and then computed the ratio $T_{CPU,cold}/T_{CPU,warm}$. As for the memory, we measured the mean percentage of RAM space used during the each experiment ($P_{RAM,cold}$ or $P_{RAM,warm}$), and then computed the ratio $P_{RAM,cold}/P_{RAM,warm}$. As clearly illustrated in the figure, the average saving in terms of resource consumption of the pod running the monitoring function, and the one of the pods running the webserver together with the database reader functions, is quite limited. It is in the order of no more than 5%, for all the values in the abscissa, but the final one, in which the data transmission period is 1.5 times $T_{idle}$. In this case, both CPU consumption and RAM usage in cold start case decrease to around 25% of warm start, thus making the cold start really convenient in a pure pay-as-you-go model. However, we also have to keep in mind the service times, which can be quite large. Thus, the usefulness of the monitoring and web query functions in cold start mode is strongly dependent on the service response time requirement. If relatively large values can be tolerated, cold start can be a viable option only if the data generation period is definitely larger than the length of the inactivity timer used to terminate an idle function. Otherwise, since latencies in cold start mode are pronouncedly larger with no significant resource saving, such a performance degradation is not justified at all. However, this is an expected behavior, since both monitoring operations and web queries in the considered scenarios are either nearly continuous or periodic. As such, they can benefit of the cold start only if the period of their invocation is significantly larger than the inactivity timer used to shut them down. Likely, what happens is that the additional overhead required to launch the function in cold start wipes out the saving associated with keeping the function off. Only when the time between the shutdown and the next restart is significant, the saving is appreciable.

A different reasoning is needed for the processing function, which is instead a pure event-based function. In our scenario, the processing function is not invoked by the monitoring function for each data packet received via MQTT, but only for those deserving more attention. Thus, the processing function is invoked by the monitoring one, only in a specific percentage of cases. In our experiment, we used a fixed probability $P_{proc}$ equal to 33%. Thus, the average period between two successive invocations of the processing function is much larger than the $T_{sensor}$ for the corresponding abscissa value. For this reason the cold start usage is always very interesting in terms of resource consumption, with a saving in the range 30–80% for the CPU and almost always higher than 50% for the memory. As for service time, we have to bear in mind that part of the delay budget of the processing service time is to ascribe to the cold start operation of the monitoring function. Depending on the service requirements, an always active monitoring function and a processing function in cold start mode could be a convenient deployment alternative, able to provide both good service time and significant resource saving.

Another interesting metric to evaluate is the number of messages to be exchanged for implementing the overall machinery, since it depends on the bandwidth needed to implement the service between pods and it gives some insight about deployment complexity. Let $\lambda_{data}=1/T_{sensor}$ denote the average arrival rate of data from sensor, and assume it to be a Poisson process. The arrival rate towards the processing function is clearly equal to $\lambda_{proc}=P_{proc}\lambda_{data}$, and it is Poisson process as well. From Figure 4, it is immediate to see that, in the steady state, in the warm case deployment option, four messages must be handled for each incoming sensor data: The one exchanged between the broker and the OpenFaaS Gateway, which re-sends the message to the monitoring function, which in turn writes data in the database, and finally returns the Gateway an Ack message. However, if the metric value evaluated by the monitoring function is larger than the threshold value, which happens with a rate $\lambda_{proc}$, it is necessary to include 3 additional messages: The messages exchanged between the monitoring and processing functions plus another writing operation into the database. Thus, it results:

(1) $$W\left(\lambda_{data}\right)=4\lambda_{data}+3\lambda_{proc}=\lambda_{data}\left(4+3P_{proc}\right).$$

As for the cold start option, for each arrival we considered also the possibility that the function that has to manage the incoming packet was off. The probability of this event is $P_{idle}^{mon}=e^{-\lambda_{data}T_{idle}}$ for the monitoring function and $P_{idle}^{proc}=e^{-\lambda_{proc}T_{idle}}$ for the processing function. Thus, following the same reasoning done for the warm start case, from the inspection of Figure 4 it results that the rate of messages to be exchanged in the cluster is equal to:

(2) $$\begin{array}{ccc} C(\lambda_{data}) & = & \lambda_{data}\left[4\left(1-P_{idle}^{mon}\right)+5P_{idle}^{mon}\right]+\lambda_{proc}\left[3\left(1-P_{idle}^{proc}\right)+5P_{idle}^{proc}\right]=\\ & = & \lambda_{data}\left\{4\left(1-P_{idle}^{mon}\right)+5P_{idle}^{mon}+P_{proc}\left[3\left(1-P_{idle}^{proc}\right)+5P_{idle}^{proc}\right]\right\}. \end{array}$$

As first hand approximation, we do not include service times in the evaluation of $C(\lambda_{data})$, whereas it has no impact on the evaluation of $W(\lambda_{data})$. However, the presence of a non negligible service time would have the only effect of making some instances of the functions in cold start potentially still running when a new request is issued, i.e., to decrease $P_{idle}^{mon}$ or $P_{idle}^{proc}$, so decreasing the difference between $C(\lambda_{data})$ and $W(\lambda_{data})$. Thus, the results shown can be regarded as an upper bound for the $C(\lambda_{data})$.

**Figure 15:**
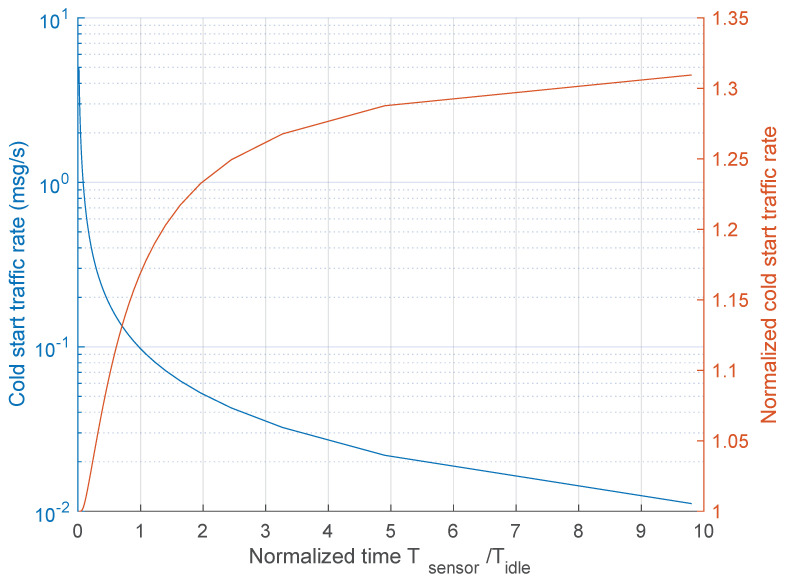
Traffic rate of the cold start mode as a function of the normalized time $T_{sensor}/T_{idle}$.

Figure 15 depicts the average number of messages per second associated with the cold start (the blue curve), that is $C(\lambda_{data})$, and its normalized version to the average traffic rate of the warm start (orange curve), that is $C\left(\lambda_{data}\right)/W\left(\lambda_{data}\right)$, as a function of the normalized interarrival time of sensor data $\frac{T_{sensor}}{T_{idle}}=\frac{1}{\lambda_{data}T_{idle}}.$ The blue curve clearly shows that the rate of messages increases up to significant values when the interarrival time is quite low, which is an expected result. In this condition, the traffic volume generated by the cold start option is nearly the same of that of the warm start option (the orange curve is quickly converging to 1). Thus, there are no issues for managing large data rates. Instead, a significant increase of traffic volume per each sensor data packet occurs when the data rate is very low. In this case, although the overhead due to the traffic volume associated with the cold start option can reach the 30% more messages, at the end it is negligible, since the overall rate is well below 1 message per second.

## 5. Conclusions and Future Work

This paper analyses the suitability of a serverless architecture for implementing services in edge computing platforms handling IoT data. The complete platform runs in Kubernetes pods and makes use of the popular OpenFaaS framework to manage function pods, offering REST APIs for controlling the interactions with the framework itself. With this test-bed, we assessed the suitability of the serverless paradigm to implement an IoT service with relatively low-rate sensor data transmission. Taking advantage of OpenFaaS flexible setup of cold and warm start mode, due to a lower inactivity time with respect to other open-source FaaS implementations [24], we compared the performance of the two corresponding deployment options of the serverless paradigm, being the aforementioned cold start and warm start, and evaluated their suitability for the considered use case. The main result is that, if latencies of few seconds can be tolerated by the application, which is realistic for different IoT use cases, the cold start paradigm is suitable. In particular, it can be used to deploy the functions that are occasionally invoked, since in this case the overhead of starting the function each time is well balanced by the saving of computing resources. This is of paramount importance in edge computing, where node capability can be limited. Instead, functions that are periodically used can only be conveniently deployed in cold start mode if the data transmission period is significantly higher than the important parameter $T_{idle}$ timer, which is the inactivity time used to trigger the shutdown of the function. Otherwise, saving of computing resources could be really modest, and does not justify the significantly increased service response time. Our future research directions in this area are essentially twofold. First of all, we are working on the definition of an analytical model based on queuing theory. This model will account for both cold and warms start behaviour and will be used to evaluate service time in dynamic conditions. In addition, we intend to carry out a performance evaluation campaign in larger settings with higher aggregated load, to investigate function scheduling in both cold and warm start mode, for minimizing resource consumption while keeping service times at acceptable levels.

## Data Availability

Not applicable.
